# Publisher Correction: Energy filtering enables macromolecular MicroED data at sub-atomic resolution

**DOI:** 10.1038/s41467-025-58078-w

**Published:** 2025-03-21

**Authors:** Max T. B. Clabbers, Johan Hattne, Michael W. Martynowycz, Tamir Gonen

**Affiliations:** 1 Howard Hughes Medical Institute, University of California, Los Angeles, CA 90095 USA; 2 Department of Biological Chemistry, University of California, Los Angeles, CA 90095 USA; 3 Department of Physiology, University of California, Los Angeles, CA 90095 USA

**Keywords:** Cryoelectron microscopy, Structural biology, Cryoelectron microscopy, Biophysics

Correction to: *Nature Communications* 10.1038/s41467-025-57425-1, published online 06 March 2025

In this article the affiliation ‘Howard Hughes Medical Institute, University of California, Los Angeles, CA 90095, USA’ was missing for Michael W. Martynowycz. The Howard Hughes affiliation has been added, and the present affiliations have been moved to the Acknowledgments section. The original article has been corrected.

