# Supplementary material for: Energy filtering enables macromolecular MicroED data at sub-atomic resolution
Source: Nat Commun. 2025 Mar 6;16:2247. doi: 10.1038/s41467-025-57425-1 (PMC11885823; doi:10.1038/s41467-025-57425-1)
Supplement: Supplementary file 1 — Supplementary text and figs [file 41467_2025_57425_MOESM1_ESM.pdf]

Supplementary Information

Energy filtering enables macromolecular MicroED data at sub-atomic resolution

Max T.B. Clabbers[1,2], Johan Hattne[1,2], Michael W. Martynowycz[2,†] and Tamir Gonen[1,2,3,*]

[1]Howard Hughes Medical Institute, University of California, Los Angeles, CA 90095
[2]Department of Biological Chemistry, University of California, Los Angeles, CA 90095
[3]Department of Physiology, University of California, Los Angeles, CA 90095

[†]Present address: Hauptman-Woodward Medical Research Institute, Buffalo, NY 14203; Department of Structural Biology, University at Buffalo, The State University of New York, Buffalo, NY 14203

*Corresponding author. E-mail address: tgonen@g.ucla.edu

List of supplementary items

Supplementary Table 1. Data processing and refinement statistics.

Supplementary Table 2. Comparison of data processing and refinement statistics of filtered and unfiltered MicroED data limited to 1.4 Å resolution.

Supplementary Figure 1. Unfiltered MicroED data.

Supplementary Figure 2. Radial averages of unfiltered and filtered MicroED data.

Supplementary Figure 3. Energy-filtered continuous rotation MicroED data.

Supplementary Figure 4. Intensity statistics for individual datasets.

Supplementary Table 1. Data processing and refinement statistics.

**Data collection**	
No. of crystals	17
Space group	$P4_32_12$
Unit cell dimensions	
a, b, c (Å)	66.92, 66.92, 107.56
α, β, γ (°)	90, 90, 90
Resolution (Å)*	56.90-1.09 (1.12-1.09)
Observed reflections	2,811,895 (95,185)
Unique reflections	98,530 (6,664)
Multiplicity	28.5 (14.3)
Completeness (%)	97.5 (90.3)
R_{merge}	0.284 (1.154)
$<I/\sigma I>$	7.8 (1.4)
$CC_{1/2}$ (%)	99.5 (33.0)
**Refinement**	
Resolution (Å)	56.82-1.09
No. of reflections	98,240
R_{work}/R_{free}	0.1498/0.1831
No. of atoms	
Protein	2,197
Ligand/ions	6
Waters	334
Hydrogen omit maps	
No. of hydrogen atoms	1,937
Peaks at ≥ 3σ	767 (39.6%)
Peaks at ≥ 2σ	1,354 (69.9%)
$<B>$ factors ($Å^2$)	
Overall	11.29
Protein	9.36
Ligand/ions	10.86
Waters	25.19
R.m.s. deviations	
Bond lengths (Å)	0.012
Bond angles (°)	1.80

*Values in parenthesis are for the highest resolution shell. Data were truncated at a mean I/σI ≥ 1.0 and a cross correlation between two random half sets that is statistically significant at $CC_{1/2}$ ~ 33%.

Supplementary Table 2. Comparison of data processing and refinement statistics of filtered and unfiltered MicroED data limited to 1.4 Å resolution.

	**Unfiltered†**	**Unfiltered#**	**Filtered**
**Data collection**			
No. of crystals	5	12	17
Rotation range (°)	63.0	20.0	20.0
Flux density ($e^-/Å^2/s$)	0.002	0.002	0.002
Fluence ($e^-/Å^2$)	1.00	0.84	0.84
Dose (MGy)‡	3.66	3.08	3.08
**Data integration**			
Space group	$P4_32_12$	$P4_32_12$	$P4_32_12$
Unit cell dimensions			
a, b, c (Å)	67.02, 67.02, 107.53	67.62, 67.62, 106.59	66.92, 66.92, 107.56
α, β, γ (°)	90, 90, 90	90, 90, 90	90, 90, 90
Resolution (Å)*	19.55-1.40 (1.45-1.40)	57.10-1.40 (1.44-1.40)	56.90-1.40 (1.44-1.40)
Observed reflections	1,231,502 (69,985)	1,101,042 (82,782)	1,371,884 (108,136)
Unique reflections	47,744 (3,442)	47,420 (3,467)	47,927 (3,494)
Multiplicity	25.8 (20.7)	23.4 (23.9)	28.6 (30.9)
Completeness (%)	97.3 (96.2)	96.0 (96.3)	98.2 (98.4)
R_{merge}	0.324 (1.784)	0.441 (1.987)	0.226 (0.598)
$<I/\sigma I>$	7.6 (1.4)	6.2 (1.4)	12.1 (5.3)
$CC_{1/2}$ (%)	99.4 (27.0)	95.9 (23.2)	99.4 (85.7)
**Refinement**			
Resolution (Å)	19.77-1.40	57.10-1.40	56.82-1.40
No. of reflections	47,738	46,737	47,907
R_{work}/R_{free}	0.1374/0.1735	0.1538/0.1856	0.1208/0.1427
No. of atoms			
Protein	2,063	2,197	2,197
Ligand/ions	10	6	6
Waters	307	334	334
$<B>$ factors ($Å^2$)			
Overall	14.47	12.19	10.61
Protein	12.51	10.11	8.52
Ligand/ions	27.44	10.15	10.19
Waters	27.21	25.92	24.39
R.m.s. deviations			
Bond lengths (Å)	0.015	0.016	0.013
Bond angles (°)	1.10	1.80	1.79

†Unfiltered MicroED data collected from 63° sweeps were previously published in Martynowycz *et al.*, 2023.

#An equivalent unfiltered MicroED dataset collected utilizing a 20° sweeps strategy, identical to the filtered data.

*Values in parenthesis are for the highest resolution shell. For an equal comparison, all data were truncated at 1.4 Å resolution.

‡ Calculated with the EMED subprogram of RADDOSE-3D (5.0.1047)[20].

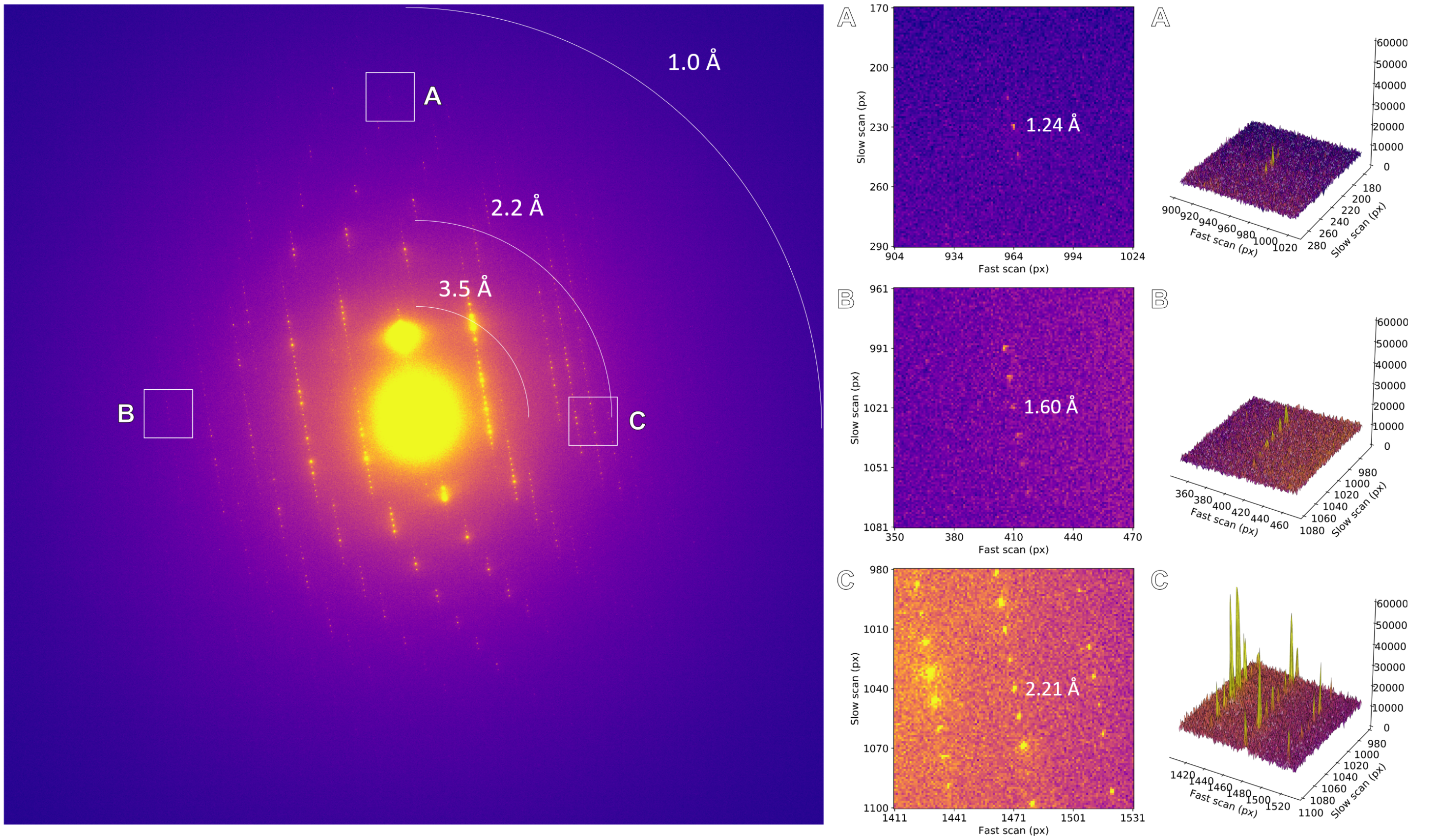

Supplementary Figure 1. Unfiltered MicroED data. Unfiltered diffraction pattern of a stationary proteinase K lamella from a 420 s exposure at a total fluence of 0.84 e⁻/Å². Highlighted areas are magnified in the right panels and the corresponding peak profiles are plotted.

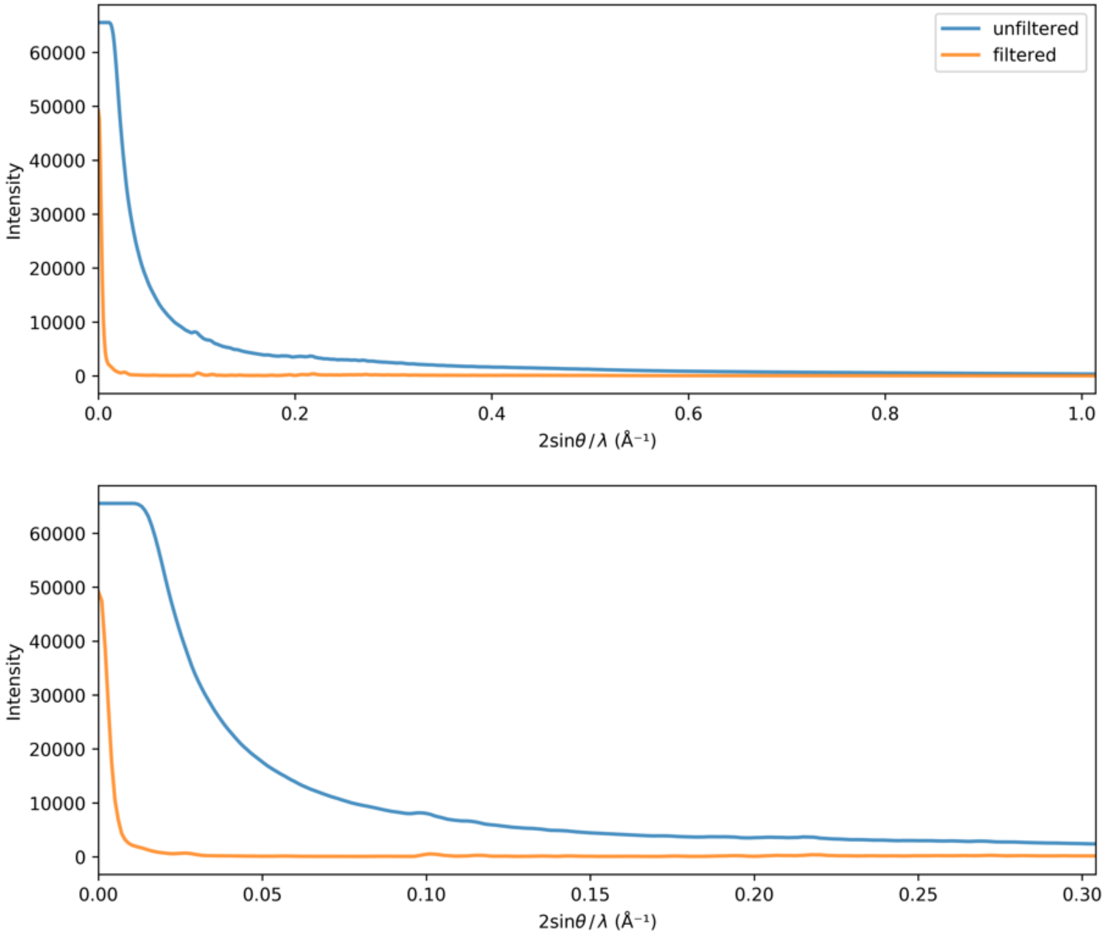

Supplementary Figure 2. Radial averages of unfiltered and filtered MicroED data. Radial averages of an energy-filtered diffraction frame (shown in Figure 1) and unfiltered data (shown in Supplementary Figure 1). Top: radial plots extending from the center peak to the edge of the detector, at approximately 1 Å resolution. Bottom: Same radial plots focusing on the lower angle data, extending to ~3 Å resolution.

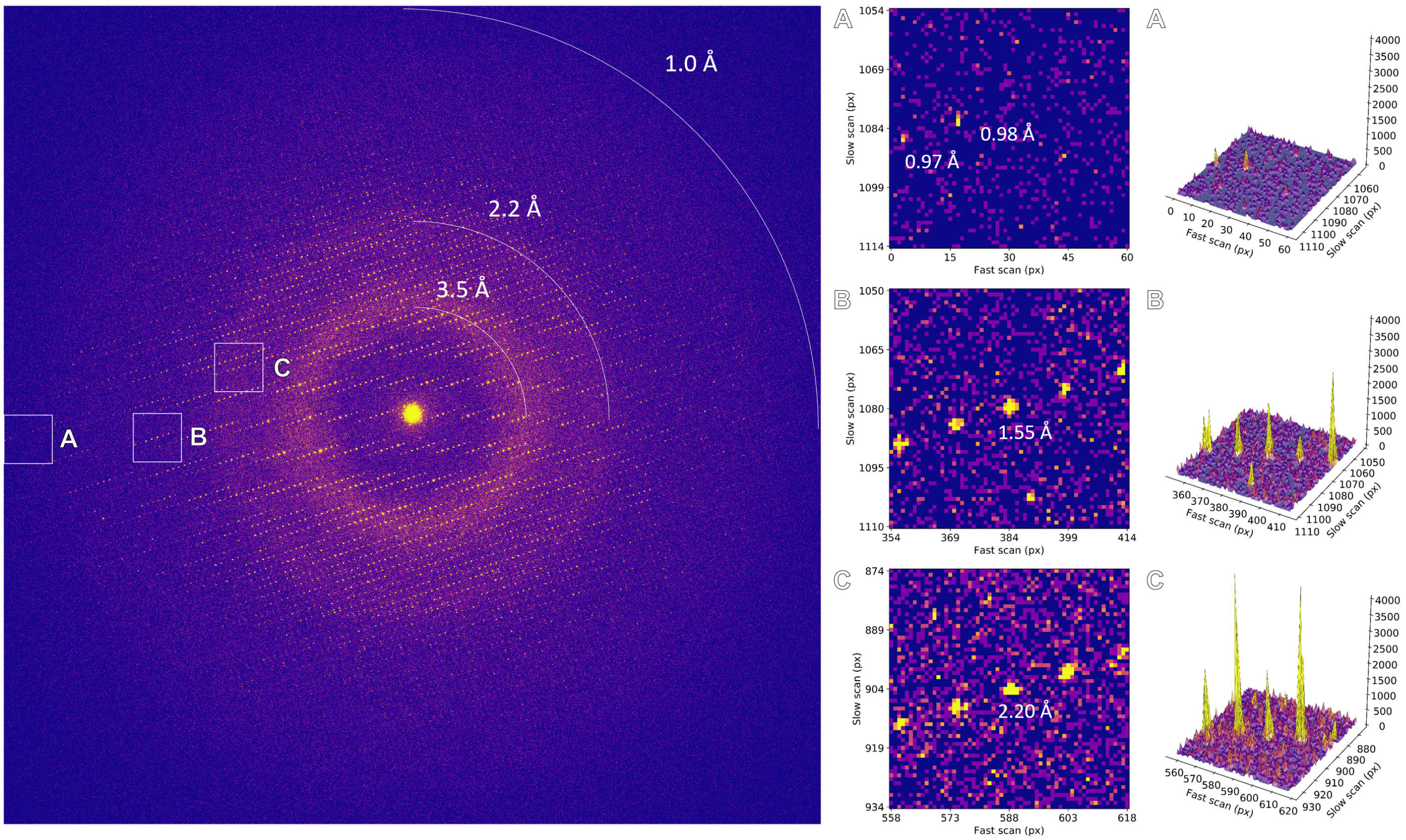

Supplementary Figure 3. Energy-filtered continuous rotation MicroED data. Initial frame from energy-filtered and electron-counted MicroED data collection with a 5 s exposure time and a total fluence of 0.01 $e^{-}/Å^{2}$. Highlighted areas are magnified in the right panels and the corresponding peak profiles are plotted.

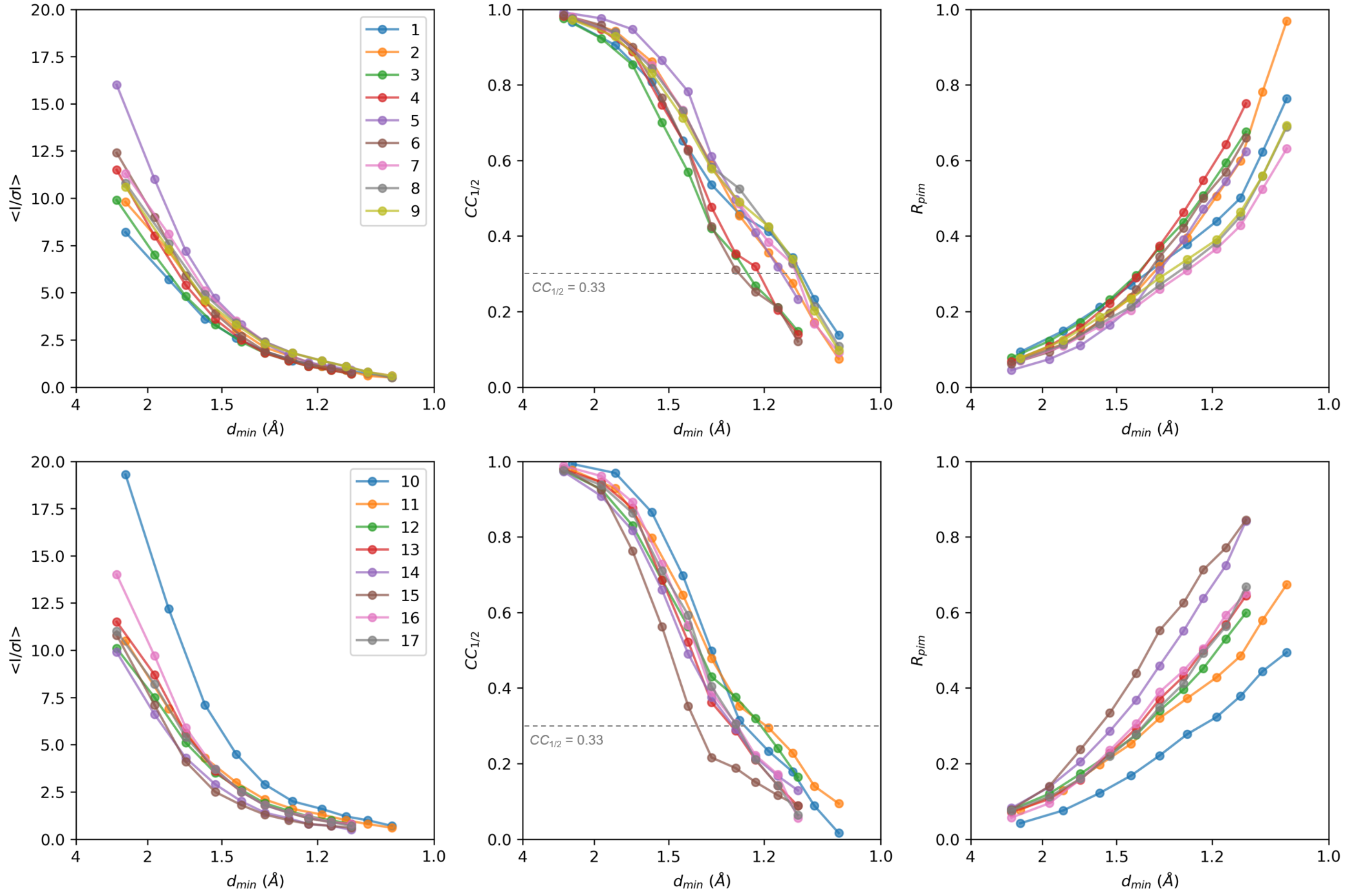

Supplementary Figure 4. Intensity statistics for individual datasets. Intensity statistics for individual MicroED datasets, featuring from left to right *I/σI*, $CC_{1/2}$, and R_{pim} as function of resolution. Individual datasets were truncated ranging from 1.13 to 1.06 Å resolution at a $CC_{1/2}$ that was still significant at the 0.1% level.
